# Facility-level CKD-MBD composite score and risk of adverse clinical outcomes among patients on hemodialysis

**DOI:** 10.1186/s12882-016-0382-8

**Published:** 2016-11-04

**Authors:** Geoffrey A. Block, Akeem A. Yusuf, Mark D. Danese, Heidi S. Wirtz, Yan Hu, Thy P. Do, Kerry Cooper, David T. Gilbertson, Brian D. Bradbury, Allan J. Collins

**Affiliations:** 1 Denver Nephrology, Denver, CO USA; 2 Chronic Disease Research Group, Minneapolis Medical Research Foundation, 914 South Eighth St, Suite S4.210, Minneapolis, MN 55404 USA; 3 Outcomes Insights, Westlake Village, CA USA; 4 Center for Observational Research, Amgen Inc, Thousand Oaks, CA USA; 5 Global Medical Organization, Amgen, Inc., Thousand Oaks, CA USA; 6 Department of Medicine, University of Minnesota, Minneapolis, MN USA

**Keywords:** Chronic kidney disease-mineral bone disorder, End-stage renal disease, Hemodialysis

## Abstract

**Background:**

Patients receiving hemodialysis with values outside of target levels for parathyroid hormone (PTH: 150–600 pg/mL), calcium (Ca: 8.4–10.2 mg/dL), and phosphate (P: 3.5–5.5 mg/dL) are at elevated morbidity and mortality risk. We examined whether patients receiving care in dialysis facilities where greater proportions of patients have at least two values out of target have a higher risk of adverse clinical outcomes.

**Methods:**

The study cohort consisted of 39,085 prevalent hemodialysis patients in 1298 DaVita dialysis facilities as of September 1, 2009, followed from January 1, 2010, until an outcome, a censoring event, or December 31, 2010. We determined the quintile of the distribution across facilities of the proportion of patients with at least two of three parameters out of, or above, target over a 4-month baseline period. The primary composite outcome was cardiovascular hospitalization or death. Secondary outcomes included death, cardiovascular hospitalization, and parathyroidectomy. Poisson regression models were used to estimate the association of facility quintile with outcomes.

**Results:**

Facility quintile was associated with a 7 % increased risk of cardiovascular hospitalization or death (quintile 5 versus 1, RR 1.07, 95 % CI 1.01–1.13) using the out-of-target measure of exposure and a 12 % increased risk (RR 1.12, 95 % CI 1.06–1.19) using the above-target measure. No association was seen for death using either measure. Patients in facility quintiles 3–5 (versus 1) were at increased parathyroidectomy risk (RR ranged from 2.05, 95 % CI 1.10–3.82, for quintile 3 to 2.73, 95 % CI 1.50–4.98, for quintile 5).

**Conclusions:**

Facility level analysis of a large prevalent sample of US patients on hemodialysis demonstrates that patients in facilities with the least control of PTH, Ca, and P had the greatest risk of parathyroidectomy or the combination of cardiovascular hospitalization or death.

**Electronic supplementary material:**

The online version of this article (doi:10.1186/s12882-016-0382-8) contains supplementary material, which is available to authorized users.

## Background

Secondary hyperparathyroidism (SHPT) is associated with a variety of adverse skeletal and cardiovascular consequences in patients with chronic kidney disease (CKD). The term “chronic kidney disease-mineral bone disorder (CKD-MBD)” replaced the older term “renal osteodystrophy” to more clearly convey the broad spectrum of sequelae that accompany abnormalities in blood levels of parathyroid hormone (PTH), calcium, and phosphate, the biochemical hallmarks of SHPT. Both highly elevated and very low concentrations of these biomarkers have consistently been associated with increased mortality risk; on the basis of these epidemiologic observations, international guidelines suggest target values for each. In addition, two recently published studies have shown that patients with uncontrolled SHPT and significant elevations of PTH, calcium, and phosphate levels may ultimately undergo surgical parathyroidectomy, the complications [1] of which have until recently been largely underappreciated [1].

Although most epidemiologic studies have investigated the prognostic importance of a single CKD-MBD value, two recent studies have focused on clinical outcomes related to the joint distribution of all three. In the first, patients were categorized into one of 36 possible phenotypes of mutually exclusive combinations of PTH, calcium, and phosphate that represent most of the excess risk associated with CKD-MBD after adjustment for baseline risk of mortality and cardiovascular hospitalization [2]. The second reported an exhaustive examination of dichotomous combinations of all possible phenotypes reflecting simultaneous achievement of zero, one, two, or all three of the target values proposed by the Kidney Disease Improving Global Outcomes (KDIGO) workgroup on CKD-MBD [3, 4]. This study found that a dichotomous construct defined by two or more out-of-target or above-range biomarkers accounted for more than 70 % of excess events (akin to sensitivity) while reducing the size of the at-risk population by at least 30 % (akin to specificity). While this was highly prognostic at the patient level, there is need to evaluate how prognostic such a simple definition is at the facility level.

In the current investigation, we aimed to extend this conceptual framework to better understand how to operationalize the patient-level CKD-MBD composite score at the facility level. This approach can be used as a starting point for discussing facility-level metrics to define quality care in CKD-MBD rather than any single parameter of PTH, calcium, or phosphate. We examined whether patients receiving care in dialysis centers where greater proportions of patients have two or more values outside of the target range for PTH, calcium, and phosphate are at increased risk for clinically meaningful outcomes related to CKD-MBD, including cardiovascular hospitalization, mortality, parathyroidectomy, and a composite endpoint of all-cause mortality and cardiovascular hospitalization.

## Methods

### Data sources

We merged two different data sources to conduct this study: the DaVita Clinical Data Warehouse and the United States Renal Data System (USRDS). Permission to merge these files was obtained from the project officers of the National Institute of Diabetes and Digestive and Kidney Diseases. We obtained laboratory data from the DaVita Clinical Data Warehouse and demographic, comorbidity, dialysis facility, hospitalization, and outcomes data from the USRDS database.

### Study population and design

We derived the study cohort from the source population of all DaVita in-center hemodialysis facilities from September 1, 2009, through December 31, 2010, reflecting the most contemporary 16-month period of data available for the linked DaVita-USRDS database.

**Fig. 1:**
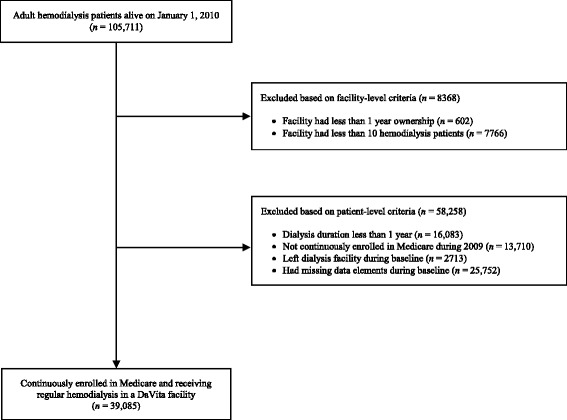
Cohort creation flowchart

Eligible facilities had at least 16 months of data during the study period to allow for a 4-month baseline period (September 1, 2009, through December 31, 2009) and a 12-month follow-up period (January 1, 2010, through December 31, 2010). Additionally, we required facilities to have had the same ownership for at least 1 year before the index date and to care for at least ten patients who met all patient-level eligibility criteria. The study cohort consisted of point prevalent hemodialysis patients derived on January 1, 2010, from the eligible facilities. Eligible patients: (1) were aged ≥ 18 years; (2) were alive on the index date; (3) were on hemodialysis for at least 1 year before the index date; (4) received care at their respective facilities during the entire 4-month baseline period; (5) were continuously enrolled in Medicare in 2009; and (6) had at least one value for each CKD-MBD-related biochemical parameter (PTH, calcium, and phosphate) during the baseline period (Fig. 1). Patients were followed from January 1, 2010 (index date), until the earliest of death, kidney transplant, change in provider or modality, Medicare disenrollment, loss to follow-up, or end of study.

### Exposure, outcome, and other measurements

The proportion of patients at each facility with at least two of three CKD-MBD biochemical parameters out of target was our primary exposure of interest. We chose this definition based on prior patient-level work by Danese et al. [3] demonstrating that at least two CKD-MBD biochemical parameters out of target best maximized identification of patients at risk of adverse clinical outcomes and minimized identification of patients not at risk. Consistent with Danese et al. [3], we considered biochemical parameters to be out of target if they were above or below the following pre-defined target ranges during the 4-month baseline period: PTH, 150–600 pg/mL; calcium, 8.4–10.2 mg/dL; phosphate, 3.5–5.5 mg/dL. For each biochemical parameter, we considered the average value over the baseline period.

We assigned each dialysis facility a single CKD-MBD composite score, calculated as the proportion of patients at that facility with at least two of three CKD-MBD biochemical parameters out of target during the baseline period. Facilities were then categorized into five groups based on quintiles of the distribution of the facility-level CKD-MBD composite score. The exposure variable for patients at each facility was defined as the quintile group for that facility. As such, patients within each facility were assigned the same quintile score, regardless of their individual, patient-level CKD-MBD composite score. Only patients who contributed to the determination of facility eligibility were considered in the calculation of the facility-level CKD-MBD composite score. However, we allowed all eligible patients at a facility to contribute events and person-time for descriptive and modeling analyses.

In secondary analyses, we evaluated two alternative exposures. First, we redefined our primary exposure using an above-target composite score, defined as the proportion of patients at each facility with at least two of three CKD-MBD biochemical parameters *above* the pre-defined target ranges during the 4-month baseline period. Second, we ascertained our exposures using a PTH range of 150–300 pg/mL to define out-of- or above-target ranges for this laboratory measure; target ranges for calcium and phosphate did not change.

The primary outcome was the first occurrence of a cardiovascular hospitalization or death. Secondary outcomes included death and parathyroidectomy, separately. The time at risk for each outcome was independently calculated for the primary endpoint and for each secondary endpoint. For cardiovascular hospitalizations, we used an algorithm from the USRDS Annual Data Report that requires the presence of one of the following primary diagnosis codes for the hospitalization: 394–398.99, 401–405, 410–420, 421.9, 422.90, 422.99, 423–438, and 440–459. For parathryoidectomy, we used a previously published approach requiring International Classification of Diseases, Ninth Revision, Clinical Modification (ICD-9-CM) procedure code 06.8x in any position on the hospital discharge claim [5].

We assessed patient-level covariates including demographic factors, time on dialysis, cause of renal failure, access type, body mass index (BMI), length of hospital stay, and comorbid conditions as of the index date. We considered a comorbid condition as being present if at least one inpatient, home health, or skilled nursing facility claim, or at least two outpatient or physician/supplier claims separated by 90 days, were found with the corresponding ICD-9-CM diagnosis code during the 12 months before the index date [6]. Facility-level covariates included facility size and geography.

### Statistical analyses

Patient characteristics and facility CKD-MBD composite score quintiles were examined using descriptive statistics for continuous (mean, standard deviation [SD]) and categorical (percentage [%]) variables.

In the primary analysis, we fitted Poisson regression models using generalized estimating equations to estimate the association between facility-level CKD-MBD composite score (categorized as quintiles) and the patient-level risk of the composite outcome (cardiovascular hospitalization or death) during the 1-year follow-up period. We used an independent correlation structure and robust standard error estimates. We provide crude and adjusted relative risk (RR) estimates and 95 % confidence intervals (CIs); models were adjusted for patient characteristics, comorbid conditions, hospital days, and facility characteristics. In secondary analyses, we fitted Poisson regression models using generalized estimating equations to estimate the association between facility-level CKD-MBD composite score and the risk of death and parathyroidectomy, separately. We evaluated this same series of relations using the above-target composite score for defining the exposure. Finally, we replicated this series of analyses (out of target and above target) using the Kidney Disease Outcomes Quality Initiative (KDOQI) definition for PTH (150–300 pg/mL). Quintile 1 (facilities with the lowest proportion of patients with out-of- or above-target values) was the reference category for all analyses.

All analyses were conducted using SAS software version 9. (Cary, NC).

## Results

**Fig. 2:**
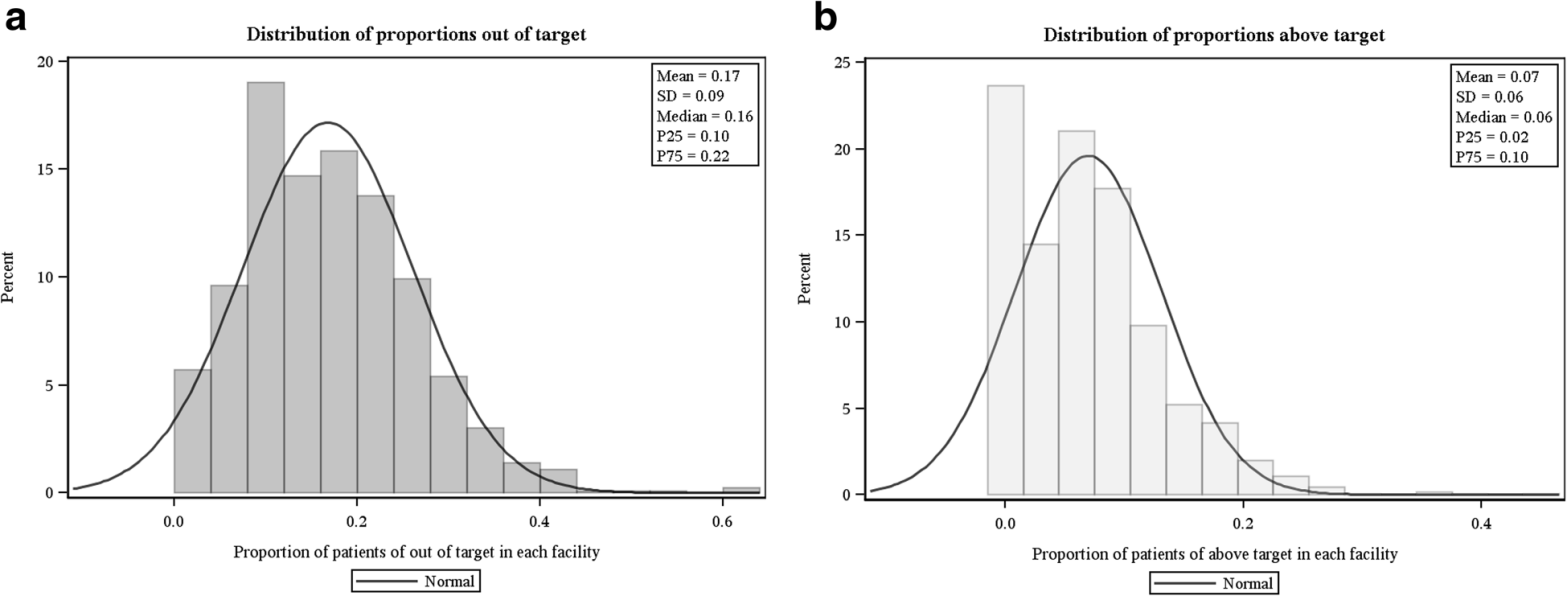
Distribution of proportion of patients with at least two of three CKD-MBD biomarkers (panel **a**) out of target ranges and (panel **b**) above target ranges. CKD-MBD, chronic kidney disease mineral bone disorder; P25, 25th percentile; P75, 75th percentile; SD, standard deviation

The final study cohort included 1298 DaVita facilities representing 39,085 patients receiving in-center hemodialysis (Fig. 1). Overall, the mean (SD) age was 62.5 (14.7) years; 54.9 % of patients were male, 51.1 % were white, and 51.5 % had been on dialysis for 4 or more years. Figures 2a and b show the distribution of proportions of patients at each facility who were out of or above target (based on individual patient-level designations). Mean (SD) facility-level proportions of patients out of target and above target were 17.2 % (8.3 %) and 7.1 % (5.5 %), respectively. At facilities in the highest quintile, representing the highest proportions of patients not achieving CKD-MBD biochemical control, proportions of patients out of target and above target were ≥ 24.5 % and ≥ 11.1 %, respectively (Table 1).

**Table 1:** Baseline characteristics, overall and by facility-level proportion of patients with at least two of three CKD-MBD biomarkers out-of- or above-target ranges, using a PTH target range of 150–600 pg/mL

		Quintile of facility-level proportion of patients with ≥ 2 out-of-target biomarkers									
		Out-of-target ranges					Above-target ranges				
	Overall	Q1: < 9.1 %	Q2: 9.1–< 13.6 %	Q3:13.6–< 18.2 %	Q4: 18.2–< 24.5 %	Q5: ≥ 24.5 %	Q1: 0 %	Q2: 0–< 4.8 %	Q3: 4.8–< 7.7 %	Q4: 7.7–< 11.1 %	Q5: ≥ 11.1 %
*n* of facilities	1298	253	259	243	283	260	303	208	262	252	273
*n* of patients	39,085	5984	7635	8515	9543	7408	6120	8045	9125	7813	7982
Age,yrs.											
18–44	12.9	10.0	11.4	12.3	14.3	15.6	10.1	11.1	11.8	14.3	16.7
45–64	40.4	35.8	36.9	41.0	42.2	44.7	35.3	38.3	40.5	41.1	45.6
65–74	24.5	28.1	25.9	24.4	23.1	22.3	28.1	25.2	24.6	24.4	21.1
≥ 75	22.2	26.1	25.8	22.3	20.5	17.4	26.4	25.4	23.1	20.2	16.5
Sex											
Male	54.9	56.4	53.9	54.4	54.9	55.4	53.9	55.2	55.0	54.4	55.7
Race											
White	51.1	56.2	57.7	50.0	49.2	44.0	63.4	57.6	55.6	43.9	37.1
Black	42.6	37.9	35.7	44.0	45.7	47.7	30.4	34.9	39.2	49.0	57.1
Other	6.3	5.9	6.5	6.0	5.0	8.3	6.1	7.5	5.1	7.1	5.8
Dialysis duration, yrs.											
< 2	18.0	21.7	20.0	18.1	15.9	15.6	23.0	19.3	17.7	16.2	15.0
2–< 4	30.5	33.3	31.9	30.9	30.0	27.0	33.5	31.3	30.5	29.1	28.6
≥ 4	51.5	44.9	48.1	51.1	54.1	57.4	43.4	49.4	51.8	54.7	56.4
ESRD cause, %											
Diabetes	45.2	48.0	46.3	45.2	43.6	43.7	48.5	46.7	45.7	44.1	41.5
Hypertension	29.7	29.7	28.4	30.6	30.6	29.0	27.5	28.7	29.6	30.2	32.1
GN	9.7	7.9	10.0	9.2	10.1	11.0	8.7	9.5	9.6	10.1	10.3
Other cause	15.4	14.3	15.4	15.0	15.7	16.3	15.3	15.1	15.0	15.5	16.0
BMI, kg/m^2^											
Mean (SD)	28.2 (7.3)	28.4 (7.4)	27.9 (7.1)	28.2 (7.4)	28.2 (7.2)	28.2 (7.3)	28.5 (7.6)	28.0 (7.0)	28.0 (7.2)	28.2 (7.3)	28.3 (7.4)
Hospital days											
0	43.8	44.9	43.7	43.7	43.5	43.7	44.2	44.6	43.4	43.9	43.1
1–4	12.5	13.0	12.3	12.7	12.4	12.1	12.2	12.7	12.2	12.7	12.4
5–10	15.5	15.2	15.5	15.1	15.8	15.9	15.2	15.6	15.5	15.8	15.4
≥ 11	28.2	26.9	28.5	28.5	28.4	28.3	28.3	27.1	28.8	27.5	29.0
Comorbidity, %											
ASHD	42.1	46.0	45.5	41.2	40.5	38.5	46.2	44.5	42.6	40.7	37.3
CHF	48.5	49.8	50.6	48.4	47.5	46.4	49.7	49.6	48.7	47.9	46.6
CVA/TIA	17.5	18.1	17.8	18.1	17.0	16.4	18.4	18.0	17.6	17.2	16.2
PVD	36.3	37.8	37.9	35.9	35.1	35.6	37.4	37.7	38.4	35.0	33.1
Other cardiac	28.1	29.1	28.2	27.9	27.7	28.0	29.8	28.2	28.0	27.3	27.6
COPD	21.4	22.1	22.2	21.5	20.9	20.6	23.1	21.2	22.6	20.0	20.3
GI bleeding	5.9	5.5	5.7	6.3	5.9	6.0	5.6	5.6	6.2	5.9	6.2
Liver disease	2.6	2.6	2.3	3.1	2.5	2.5	2.6	2.3	2.8	2.8	2.5
Dysrhythmia	25.3	26.4	27.9	25.1	24.7	23.0	27.2	25.8	25.6	25.0	23.5
Cancer	9.1	10.5	9.5	9.3	8.2	8.2	10.2	9.5	9.5	8.6	7.8
Diabetes	63.4	65.8	64.8	64.1	62.4	60.7	67.0	64.5	64.3	62.0	60.0
Previous CV hospitalization	25.3	25.0	25.6	25.9	25.3	24.6	24.8	25.2	25.4	24.8	26.2
Previous PTX	0.7	0.4	0.5	0.7	0.7	1.1	0.5	0.6	0.6	0.8	0.7
Laboratory values, mean (SD)^a^											
PTH, pg/mL	341.7 (320.3)	294.2 (221.0)	308.7 (255.5)	339.0 (308.7)	361.9 (358.3)	391.1 (392.2)	273.3 (170.2)	296.7 (233.8)	326.1 (253.4)	368.1 (353.3)	431.3 (435.8)
Calcium, mg/dL	9.0 (0.6)	9.1 (0.5)	9.0 (0.5)	9.0 (0.5)	9.0 (0.6)	8.9 (0.7)	9.0 (0.51)	9.0 (0.5)	9.0 (0.6)	9.0 (0.6)	9.0 (0.6)
Phosphate, mg/dL	5.3 (1.3)	5.1 (1.2)	5.2 (1.2)	5.3 (1.3)	5.3 (1.4)	5.4 (1.4)	5.1 (1.2)	5.2 (1.3)	5.3 (1.3)	5.3 (1.3)	5.5 (1.4)

Quintiles of facility-level proportion of CKD-MBD composite score were based on proportions of patients at each facility whose values were out of or above target. “Out of target” was characterized by at least two CKD-MBD laboratory values above or below defined target ranges for PTH, calcium, and phosphate. “Above target” was characterized by at least two CKD-MBD laboratory values above defined target ranges for PTH, calcium, and phosphate. Target ranges for CKD-MBD laboratory values were: 150–600 pg/mL for PTH, 8.4–10.2 mg/dL for calcium, and 3.5–5.5 mg/dL for phosphate

*Abbreviations*: *ASHD* atherosclerotic heart disease; *BMI* body mass index; *CHF* congestive heart failure; *CKD-MBD* chronic kidney disease- mineral bone disorder; *COPD* chronic obstructive pulmonary disease; *CV* cardiovascular; *CVA/TIA* cerebrovascular accident/transient ischemic attack; *ESRD* end-stage renal disease; *GI* gastrointestinal; *GN* glomerulonephritis; *PTH* parathyroid hormone; *PTX* parathyroidectomy; *PVD* peripheral vascular disease; *Q* quintile; *SD* standard deviation

^a^Mean laboratory values ascertained during the 4-month baseline period

Patients at facilities in the higher quintiles of the CKD-MBD out-of-target composite score were more likely to be younger, to be black, and to have less comorbidity, longer time on dialysis, a history of parathyroidectomy, and higher baseline PTH and phosphate levels (Table 1). Characteristics by facility-level CKD-MBD above-target composite score are also shown in Table 1; patterns of baseline characteristics did not differ appreciably from the CKD-MBD out-of-target composite score. Patterns of baseline characteristics also did not change meaningfully when we ascertained our exposures for CKD-MBD out-of- or above-target composite scores using a PTH range of 150–300 pg/mL rather than 150–600 pg/mL (Additional file 1: Table S1).

**Fig. 3:**
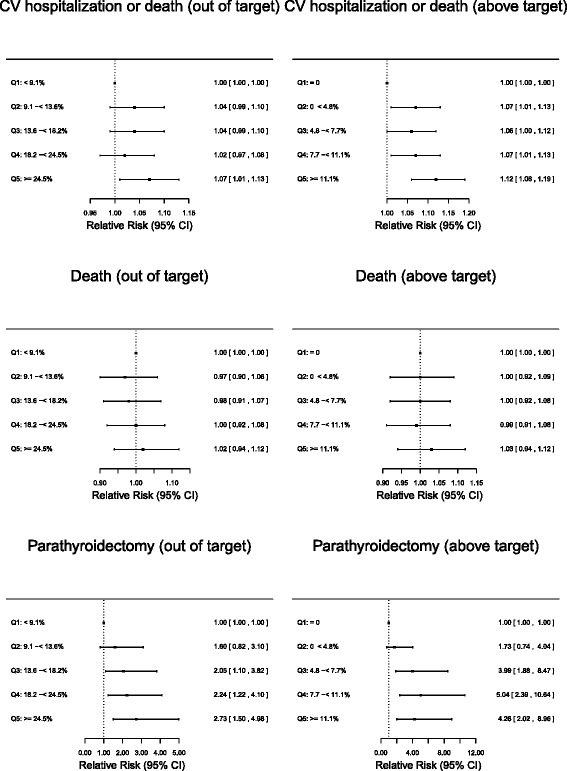
Relative risks and 95 % confidence intervals for risk of adverse clinical events associated with quintile of facility-level proportion of patients with at least two of three CKD-MBD biomarkers out of or above target ranges, using a PTH target range of 150–600 pg/mL. Quintiles of facility-level CKD-MBD composite score were based on proportions of patients at each facility who were out of or above target. “Out of target” was characterized by at least two CKD-MBD laboratory values above or below defined target ranges for PTH, calcium, and phosphate. “Above target” was characterized by at least two CKD-MBD laboratory values above defined target ranges for PTH, calcium, and phosphate. Target ranges for CKD-MBD laboratory values were 150–600 pg/mL for PTH, 8.4–10.2 mg/dL for calcium, and 3.5–5.5 mg/dL for phosphate. Analyses were adjusted for baseline patient demographics, dialysis duration, previous hospitalization, body mass index, comorbid conditions, facility size, and geography. CKD-MBD, chronic kidney disease-mineral bone disorder; CV, cardiovascular; CI confidence interval; PTH, parathyroid hormone

In the multivariable adjusted models, the CKD-MBD composite score was associated with a 7 % elevated risk comparing quintile 5 to quintile 1 (RR 1.07, 95 % CI 1.01–1.13); there was no association for quintiles 2–4 (Fig. 3). When we used the above-target composite score as the measure of exposure, we found that patients in quintile 5 were again at higher risk of cardiovascular hospitalization or death compared with patients in quintile 1 (RR 1.12, 95 % CI 1.06–1.19), and patients in quintiles 2–4 were also at higher risk (RR 1.07, 95 % CI 1.01–1.13 for quintile 2; RR 1.06, 95 % CI 1.00–1.12 for quintile 3; RR 1.07, 95 % CI 1.01–1.13 for quintile 4).

For the individual outcome of death, we found no associations with facility-level CKD-MBD out-of- or above-target composite score quintiles. For parathyroidectomy, patients in quintiles 3–5 were at elevated risk compared with patients in quintile 1 (RR 2.05, 95 % CI 1.10–3.82 for quintile 3; RR 2.24, 95 % CI 1.22–4.10 for quintile 4; and RR 2.73, 95 % CI 1.50–4.98 for quintile 5). Results were similar when the facility-level CKD-MBD above-target composite score was used as the measure of exposure, though the magnitude of event risk was higher.

Results from sensitivity analyses using the more conservative definition of 150–300 pg/mL rather than 150–600 pg/mL as the the target range for PTH were consistent with the main analyses (Additional file 2: Figure S1).

## Discussion

In this study of nearly 1300 US dialysis centers and 39,000 patients receiving in-center hemodialysis between 2009 and 2011, we characterized all facilities using a facility-level CKD-MBD composite score defined by at least two of three biochemical parameters out of target, or separately, above target. A fifth of all facilities had ≥ 24 % of their patients out of target and ≥ 11 % of their patients above target. Using this composite score, we conducted a facility-level analysis and found that patients receiving care at facilities with higher composite scores were at elevated risk of adverse clinical outcomes compared with patients receiving care at facilities with the lowest composite scores. The increased risk was nearly 15 % for cardiovascular hospitalization or death over a 1-year period and ~300 % for parathyroidectomy. The results were more pronounced when the analysis was conducted using a composite score defined by above-target values. These data provide additional evidence that characterizing CKD-MBD using a construct that classifies patients simultaneously on all three biochemical parameters is achievable, has significant prognostic value, and may be a useful framework for identifying clinically meaningful differences in severity of CKD-MBD.

The results of this study extend previous patient-level analyses showing that two or more of the three biochemical hallmarks of CKD-MBD, PTH, calcium, and phosphate, out of or above the target ranges established by KDOQI or Kidney Disease: Improving Global Outcomes (KDIGO) strongly predicts the risk of major clinical outcomes, independent of case-mix and baseline comorbidity [2, 3, 7]. In addition, these results support the consistently observed independent relationships between individual biochemical variables and clinical outcomes [8–10] and of robust pre-clinical literature describing the adverse effects of exposure of vascular cells to media containing high phosphate and/or high calcium [11, 12]. These findings also provide important context for the Evaluation of Cinacalcet Hydrochloride Therapy to Lower Cardiovascular Events (EVOLVE) trial findings; EVOLVE, a randomized, double blind placebo-controlled clinical study [13], was designed to test whether treatment with cinacalcet in addition to standard of care in patients with SHPT receiving hemodialysis would reduce the risk of death and cardiovascular events relative to placebo plus standard of care. The primary unadjusted intention-to-treat analysis demonstrated reductions in PTH, calcium, and phosphate, and a greater than 50 % reduction in risk of parathyroidectomy for patients randomized to cinacalcet, but a non-definitive reduction in the primary composite end point of death or cardiovascular events (hazard ratio, 0.93; 95 % CI, 0.85–1.02). Post-hoc analyses adjusting for the age imbalance in the treatment groups despite randomization showed nominally significant reduction in the primary composite end point (death or cardiovascular events), providing further evidence of the potential benefit of lowering PTH, calcium, and phosphate levels. The current facility-level analysis findings reinforce the strong link between SHPT management (as reflected in facility-level target achievement) and subsequent clinical outcome risk, parathyroidectomy in particular.

Complex inter-relationships between PTH, calcium, and phosphate are such that a change in any one of them is accompanied by changes in the others, often in opposite directions. Considering any in isolation of the others (as analyses evaluating the independent effects of one adjusting for the others would inform) is inconsistent with the clinical situations physicians encounter. It is exceedingly common for clinicians to encounter scenarios in which clinical guidelines seemingly give contradictory guidance relative to these three biochemical measures of quality of care, and providers must decide the relative risks of achieving one particular goal while potentially abandoning another. In addition, therapy directed at a single measure often has unintended consequences (direct and indirect) on the others that must be addressed [14, 15]. In such a complex clinical situation, a composite measure that incorporates PTH, calcium, and phosphate can help physicians identify patients at greatest risk of adverse outcomes who might benefit most from therapeutic intervention. In the current investigation, we aggregated the previously identified patient-level composite score into a facility-level measure and showed similar significant prognostic value, reinforcing the construct validity of this measure as a reliable marker of clinically meaningful CKD-MBD.

While our facility-level study provides a simple, integrated approach to identify facilities at highest risk of adverse clinical outcomes, we did not address directly the means by which different dialysis centers achieve lower proportions of out-of-target patients. Management of SHPT is complicated and the strategies employed by dialysis providers in clinical practice are not easily defined, aside from calculating average utilization, which by itself does not accurately capture the complex interplay of the various therapeutic interventions. Understanding which therapeutic approaches enable improved composite biochemical control without increasing safety risks will be an important benefit/risk calculation. For example, using aluminum as a phosphate binder is a therapeutic approach that could simultanesouly reduce both PTH and phosphate and might enable greater composite biochemical control; however, this would be at the expense of patient safety. An instructive contemporary example is the recent use of lower dialysate calcium concentrations in dialysis centers. A large non-interventional study using dialysis provider data found that patients receiving care in centers that predominantly use a < 2.5 mEq/L dialysate calcium concentration (compared with patients at centers using a ≥ 2.5 mEq/L concentration) achieved less biochemical control and were at elevated risk of heart failure hospitalization, intradialytic hypotension, and hypocalcemia [16]. Some of these decisions could be motived by the economic pressures introduced by the prospective payment system encouraging use of lower cost treatments, but they should be guided by efficacy and safety data obtained from rigorously conducted clinical trials or high-quality non-experimental research conducted in large populations of patients on dialysis.

This study should be evaluated in light of the following limitations: Our primary analyses used a PTH target range of 150–600 pg/mL to reflect current clinical practice guidelines despite prior patient-level work demonstrating that a PTH target range of 150–300 pg/mL also provides significant discrimination [3]. Although the analyses were conducted at the facility level and accounted for various patient risk factors, we cannot exclude the possibility of residual confounding. However, it should be acknowledged that the adjustments made only attenuated the observed effect estimates modestly, suggesting the findings are fairly robust. Our modeling effort did not account for other facility practices or quality of care indicators (e.g., standardized mortality ratio), so it is not possible to conclude definitively from our analysis that the CKD-MBD score reflects a facility’s quality of care related only to CKD-MBD. However, our models do account for a wide range of clinical prognostic indicators that have been used extensively in studies of dialysis (e.g., vascular access, comorbid conditions such as congestsive heart failure, peripheral vascular disease, cancer). Although there may be some residual error, it is unlikely that it would materially affect the study’s conclusions. For example, the clear relation between higher CKD-MBD quintile and risk of parathyroidectomy is unlikely to be affected by a facility’s mortality performance over a calendar year. The assessment of CKD-MBD biochemical variables was conducted during a 4-month baseline period; thus, up to four values were available for calcium and phosphate, but likely only one or two values were available for PTH. If those values represent spurious measurements, our findings likely underestimate the true risk of out-of-target values. As noted, our study did not address the mechanisms facilities used to achieve improved biochemical outcomes, and we are unable to comment on whether any single treatment strategy that modifies multiple biochemical targets may be associated with benefit or harm. Clinical trials randomizing patients to different treatment protocols would provide the most reliable evidence to address those questions.

## Conclusion

In conclusion, we conducted a facility-level analysis of a large sample of US patients on dialysis and their associated dialysis facilities and found that patients at centers with the highest CKD-MBD composite scores had the greatest risk of major adverse clinical outcomes, specifically parathyroidectomy, or the combination of cardiovascular hospitalization or death. These findings provide additional evidence that a composite score accounting for all three biochemical hallmarks of SHPT (PTH, Ca, and P) has prognostic value for patients on dialysis.

## Additional files

Additional file 1: Baseline characteristics overall and by facility-level proportions of patients with at least two of three CKD-MBD biomarkers out of or above target ranges, using a PTH target range of 150–300 pg/mL. CKD-MBD, chronic kidney disease-mineral bone disorder; PTH, parathyroid hormone. (PDF 62 kb)
Additional file 2: Figure S1. Relative risks and 95 % confidence intervals for risk of adverse clinical events associated with quintile of facility-level proportions of patients with at least two of three CKD-MBD biomarkers out of or above target ranges, using a PTH target range of 150–300 pg/mL. CKD-MBD, chronic kidney disease-mineral bone disorder; PTH, parathyroid hormone. (PDF 67 kb)

## Abbreviations

BMI: Body mass index
CI: Confidence interval
CKD: Chronic kidney disease
EVOLVE: Evaluation of Cinacalcet Hydrochloride Therapy to Lower Cardiovascular Events
ICD-9-CM: International Classification of Diseases, Ninth Revision, Clinical Modification
KDIGO: Kidney Diseare: Improving Global Outcomes
KDOQI: Kidney Disease Outcomes Quality Initiative
MBD: Mineral bone disorder
PTH: Parathyroid hormone
RR: Relative risk
SD: Standard deviation
SHPT: Secondary hyperthyroidism
USRDS: United States Renal Data System

## References

[1] Ishani A, Liu J, Wetmore JB, Lowe KA, Do T, Bradbury BD (2015). Clinical outcomes after parathyroidectomy in a nationwide cohort of patients on hemodialysis. Clin J Am Soc Nephrol.

[2] Block GA, Kilpatrick RD, Lowe KA, Wang W, Danese MD (2013). CKD-Mineral and Bone Disorder and Risk of Death and Cardiovascular Hospitalization in Patients on Hemodialysis. Clin J Am Soc Nephrol.

[3] Danese MD, Halperin M, Lowe KA, Bradbury BD, Do TP, Block GA (2015). Refining the definition of clinically important mineral and bone disorder in hemodialysis patients. Nephrol Dial Transplant.

[4] Kidney Disease: Improving Global Outcomes (KDIGO) CKD-MBD Workgroup. KDIGO clinical practice guideline for the diagnosis, evaluation, prevention, and treatment of Chronic Kidney Disease-Mineral and Bone Disorder (CKD-MBD). Kidney Int. 2009;76(Suppl 113):S1–130. 10.1038/ki.2009.188 19644521

[5] Li S, Chen YW, Peng Y, Foley RN, St Peter WL (2011). Trends in parathyroidectomy rates in US hemodialysis patients from 1992 to 2007. Am J Kidney Dis.

[6] Hebert PL, Geiss LS, Tierney EF, Engelgau MM, Yawn BP, McBean AM (1999). Identifying persons with diabetes using Medicare claims data. Am J Med Qual.

[7] Danese MD, Belozeroff V, Smirnakis K, Rothman KJ (2008). Consistent control of mineral and bone disorder in incident hemodialysis patients. Clin J Am Soc Nephrol.

[8] Kalantar-Zadeh K, Block G, Humphreys MH, McAllister CJ, Kopple JD (2004). A low, rather than a high, total plasma homocysteine is an indicator of poor outcome in hemodialysis patients. J Am Soc Nephrol.

[9] Block GA, Klassen PS, Lazarus JM, Ofsthun N, Lowrie EG, Chertow GM (2004). Mineral metabolism, mortality, and morbidity in maintenance hemodialysis. J Am Soc Nephrol.

[10] Fernandez-Martin JL, Martinez-Camblor P, Dionisi MP, Floege J, Ketteler M, London G, COSMOS group (2015). Improvement of mineral and bone metabolism markers is associated with better survival in haemodialysis patients: the COSMOS study. Nephrol Dial Transplant.

[11] Shroff R, Long DA, Shanahan C (2013). Mechanistic insights into vascular calcification in CKD. J Am Soc Nephrol.

[12] Shanahan CM (2013). Mechanisms of vascular calcification in CKD-evidence for premature ageing?. Nat Rev Nephrol.

[13] Chertow GM, Block GA, Correa-Rotter R, Drüeke TB, Floege J, Goodman WG (2012). Effect of cinacalcet on cardiovascular disease in patients undergoing dialysis. N Engl J Med.

[14] Teng M, Wolf M, Lowrie E, Ofsthun N, Lazarus JM, Thadhani R (2003). Survival of patients undergoing hemodialysis with paricalcitol or calcitriol therapy. N Engl J Med.

[15] Li J, Molnar MZ, Zaritsky JJ, Sim JJ, Streja E, Kovesdy CP (2013). Correlates of parathyroid hormone concentration in hemodialysis patients. Nephrol Dial Transplant.

[16] Brunelli SM, Sibbel S, Do TP, Cooper K, Bradbury BD (2015). Facility dialysate calcium practices and clinical outcomes among patients receiving hemodialysis: a retrospective observational study. Am J Kidney Dis.

