# Supplementary material for: Facility-level CKD-MBD composite score and risk of adverse clinical outcomes among patients on hemodialysis
Source: BMC Nephrol. 2016 Nov 4;17:166. doi: 10.1186/s12882-016-0382-8 (PMC5097438; doi:10.1186/s12882-016-0382-8)
Supplement: Additional file 1: — Baseline characteristics overall and by facility-level proportions of patients with at least two of three CKD-MBD biomarkers out of or above target ranges, using a PTH target range of 150–300 pg/mL. CKD-MBD, chronic kidney disease-mineral bone disorder; PTH, parathyroid hormone. (PDF 62 kb) [file 12882_2016_382_MOESM1_ESM.pdf]

Table S1. Baseline characteristics overall and by facility-level proportions of patients with at least two of three CKD-MBD biomarkers out of or above target ranges, using a PTH target range of 150-300 pg/mL

		Quintile of Facility-Level Proportion of Patients with ≥ 2 Out-of-Target Biomarkers									
		Out-of-Target Ranges					Above-Target Ranges				
	Overall	Q1: < 21.1%	Q2: 21.1- < 27.8%	Q3: 27.8- < 33.3%	Q4: 33.3- < 40.0%	Q4: ≥ 40.0%	Q1: < 11.8%	Q2: 11.8- < 17.4%	Q3: 17.4- < 21.7%	Q4: 21.7- < 27.9%	Q5: ≥ 27.9%
n of facilities	1,298	258	256	243	266	275	250	262	266	260	260
n of patients	39,085	6,074	8,012	8,236	9,220	7,543	5,962	8,633	8,552	8,421	7,517
Age, yrs.											
18-44	12.9	9.7	11.0	12.2	14.4	16.4	9.4	10.6	12.3	15.1	16.5
45-64	40.4	33.9	38.0	40.1	42.8	45.6	34.4	39.2	39.5	41.9	45.9
65-74	24.5	27.1	26.0	24.9	23.5	21.8	26.9	25.7	24.8	23.9	21.7
≥ 75	22.2	29.4	25.0	22.8	19.3	16.2	29.2	24.5	23.4	19.1	15.9
Sex											
Male	54.9	55.6	55.6	54.0	53.7	56.0	54.9	54.6	54.8	55.1	55.1
Race											
White	51.1	63.4	56.4	52.3	47.4	39.0	62.2	58.9	53.2	45.0	37.9
Black	42.6	29.8	37.3	40.8	47.0	55.0	30.9	34.0	40.5	49.5	56.2
Other	6.3	6.8	6.3	6.9	5.6	6.1	6.9	7.1	6.2	5.5	5.9
Dialysis duration, yrs.											
< 2	18.0	21.7	20.3	17.9	15.9	15.3	22.0	18.7	18.7	15.4	16.1
2-< 4	30.5	33.1	31.6	30.3	29.8	28.2	32.8	30.1	30.3	30.7	29.1
≥ 4	51.5	45.1	48.1	51.8	54.3	56.6	45.2	51.2	51.1	53.9	54.8
ESRD cause, %											
Diabetes	45.2	46.3	47.3	46.0	44.8	41.6	45.9	48.3	46.6	42.1	42.8
Hypertension	29.7	28.9	28.4	30.0	29.5	31.9	28.5	28.3	27.9	31.6	32.2
GN	9.7	9.2	9.1	8.9	10.3	10.9	9.7	8.8	9.6	10.5	10.0
Other cause	15.4	15.6	15.2	15.1	15.5	15.6	16.0	14.6	15.8	15.8	14.9
Unknown	43.9	37.7	40.6	44.6	45.9	49.5	37.9	43.9	43.4	45.7	47.4
BMI, kg/m^2											
Mean (SD)	28.2 (7.3)	27.9 (7.1)	28.1 (7.1)	28.2 (7.3)	28.3 (7.4)	28.3 (7.5)	28.0 (7.2)	28.0 (7.0)	28.1 (7.3)	28.3 (7.4)	28.4 (7.5)
Hospital days											

0	43.8	45.5	43.4	43.4	43.8	43.4	45.6	43.9	42.2	45.0	42.8
1-4	12.5	12.5	12.7	12.2	12.3	12.8	11.9	12.5	12.2	12.5	13.1
5-10	15.5	15.1	15.8	15.5	15.5	15.5	15.7	15.4	16.4	15.2	14.9
≥ 11	28.2	26.9	28.1	28.9	28.4	28.3	26.8	28.2	29.1	27.3	29.3
Comorbidity, %											
ASHD	42.1	47.5	44.2	42.2	40.0	38.0	47.1	43.0	44.6	38.6	38.2
CHF	48.5	49.3	50.9	47.7	48.1	46.4	49.3	49.4	50.2	45.8	47.6
CVA/TIA	17.5	17.3	17.8	17.9	17.6	16.6	18.0	17.6	17.9	16.4	17.4
PVD	36.3	38.6	38.0	35.8	36.1	33.7	37.9	37.0	38.0	35.6	33.1
Other cardiac	28.1	29.0	29.3	27.2	27.2	28.3	28.6	27.8	29.5	27.3	27.4
COPD	21.4	22.0	21.7	21.7	21.0	20.7	21.5	22.3	22.1	20.2	20.9
GI bleeding	5.9	5.4	5.7	6.2	6.1	6.1	5.6	5.7	6.4	5.6	6.1
Liver disease	2.6	2.7	2.9	2.3	2.7	2.5	2.5	3.0	2.6	2.6	2.3
Dysrhythmia	25.3	27.9	26.2	25.8	24.0	23.5	27.8	24.9	26.9	23.8	23.9
Cancer	9.1	10.1	9.9	9.5	8.3	7.7	9.6	9.7	9.7	8.7	7.6
Diabetes	63.4	64.7	66.3	63.7	62.7	60.0	64.4	66.1	65.0	60.8	60.8
Previous CV hospitalization	25.3	24.3	25.7	26.1	25.4	24.8	24.0	24.9	26.5	24.5	26.4
Previous PTX	0.7	0.4	0.4	0.8	0.7	1.0	0.4	0.6	0.6	0.9	0.7
Laboratory values, mean (SD) [a]											
Phosphate, mg/dl	5.3(1.3)	5.1 (1.2)	5.2 (1.3)	5.3 (1.3)	5.3 (1.3)	5.5 (1.4)	5.0 (1.2)	5.1 (1.3)	5.3 (1.3)	5.4 (1.3)	5.5 (1.4)
Calcium, mg/dl	9.0 (0.6)	9.1 (0.5)	9.0 (0.5)	9.0 (0.6)	9.0 (0.6)	8.9 (0.6)	9.0 (0.5)	9.0 (0.5)	9.0 (0.6)	9.0 (0.6)	9.0 (0.6)
PTH, pg/ml	341.7 (320.3)	287.8 (250.1)	311.7 (267.0)	331.2 (301.0)	360.6 (343.2)	405.2 (393.4)	274.8 (214.3)	308.5 (265.2)	333.8 (320.3)	367.6 (360.4)	412.7 (378.6)

Quintiles of facility level proportion of CKD-MBD composite score was based on proportions of patients at each facility who were out of or above target. “Out of target” was characterized by at least two CKD-MBD laboratory values above or below defined target ranges for PTH, calcium, and phosphate. “Above target” was characterized by at least two CKD-MBD laboratory values above defined target ranges for PTH, calcium, and phosphate. Target ranges for CKD-MBD laboratory values were 150-300 pg/ml for PTH, 8.4-10.2 mg/dL for calcium, and 3.5-5.5 mg/dL for phosphate.

Abbreviations: ASHD, atherosclerotic heart disease; BMI, body mass index; CHF, congestive heart failure; CKD-MBD, chronic kidney disease-mineral bone disorder; COPD, chronic obstructive pulmonary disease; CV, cardiovascular; CVA/TIA, cerebrovascular accident/transient ischemic attack; ESRD, end-stage renal disease; GI gastrointestinal; PTH, parathyroid hormone; PTX, parathyroidectomy; PVD, peripheral vascular disease; Q, quintile; SD, standard deviation.

[a] Mean laboratory values ascertained during the 4-month baseline period.
