# Supplementary material for: Facility-level CKD-MBD composite score and risk of adverse clinical outcomes among patients on hemodialysis
Source: BMC Nephrol. 2016 Nov 4;17:166. doi: 10.1186/s12882-016-0382-8 (PMC5097438; doi:10.1186/s12882-016-0382-8)
Supplement: Additional file 2: Figure S1. — Relative risks and 95 % confidence intervals for risk of adverse clinical events associated with quintile of facility-level proportions of patients with at least two of three CKD-MBD biomarkers out of or above target ranges, using a PTH target range of 150–300 pg/mL. CKD-MBD, chronic kidney disease-mineral bone disorder; PTH, parathyroid hormone. (PDF 67 kb) [file 12882_2016_382_MOESM2_ESM.pdf]

Figure S1. Relative risks and 95% confidence intervals for risk of adverse clinical events associated with quintile of facility-level proportions of patients with at least two of three CKD-MBD biomarkers out of or above target ranges, usinga PTH target range of 150-300 pg/mL.

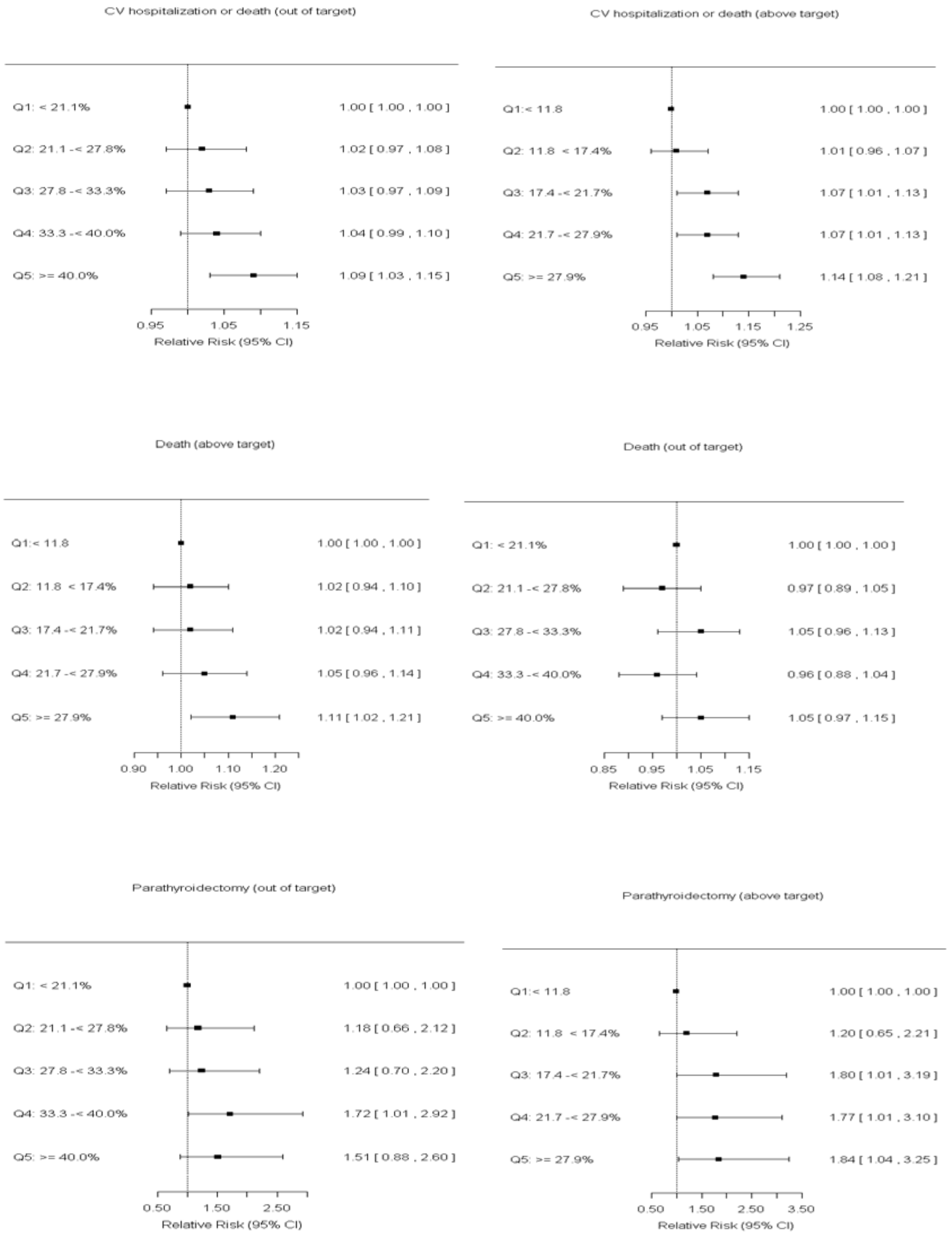

Quintiles of facility level proportions of CKD-MBD composite score were based on proportions of patients within each facility who were out of target or above target. “Out of target” was characterized by at least two CKD-MBD laboratory values above or below defined target ranges for PTH, calcium, and phosphate. “Above target” was characterized by at least two CKD-MBD laboratory parameters above defined target ranges for PTH, calcium, and phosphate. Target ranges for CKD-MBD laboratory parameters were 150-300 pg /mL for PTH, 8.4-10.2 mg/dL for calcium, and 3.5-5.5 mg/dL for phosphate. Analyses were adjusted for baseline patient demographics, dialysis duration, previous hospitalization, body mass index, comorbid conditions, facility size, and geography. CKD-MBD, chronic kidney disease-mineral bone disorder; CV, cardiovascular; CI confidence interval; PTH, parathyroid hormone.
